# Anti-Melanogenic Effects of Flavonoid Glycosides from *Limonium tetragonum* (Thunb.) Bullock via Inhibition of Tyrosinase and Tyrosinase-Related Proteins

**DOI:** 10.3390/molecules22091480

**Published:** 2017-09-05

**Authors:** Seul-Gi Lee, Fatih Karadeniz, Youngwan Seo, Chang-Suk Kong

**Affiliations:** 1 Department of Food and Nutrition, College of Medical and Life Sciences, Silla University, Baegyang-dero 700beon-gil 140, Sasang-gu, Busan 46958, Korea; tmfrl339@naver.com; 2 Marine Biotechnology Center for Pharmaceuticals and Foods, Silla University, Baegyang-dero 700beon-gil 140, Sasang-gu, Busan 46958, Korea; karadenizf@outlook.com; 3 Division of Marine Bioscience, College of Ocean Science and Technology, Korea Maritime and Ocean University, Busan 49112, Korea; ywseo@kmou.ac.kr; 4 Department of Convergence Study on the Ocean Science and Technology, Ocean Science and Technology School, Korea Maritime and Ocean University, Busan 49112, Korea

**Keywords:** cell culture, chemical analysis, colour cosmetics, *Limonium tetragonum*, melanogenesis

## Abstract

Overproduction and stimulation of tyrosinase result in increased melanogenesis of which several skin disorders such as freckles, spots, and hyperpigmentation appear as complications. *Limonium tetragonum* is a halophyte well-known for its antioxidative properties. This study investigated the anti-melanogenic effects of solvent-partitioned *L. tetragonum* extracts (LTEs) and its bioactive constituents, two isolated flavonoid glycosides. Current study followed a set of experiments on B16-F10 mouse melanoma cell model with a focus on tyrosinase activity and production. The anti-melanogenic capacity of LTEs was confirmed by their tyrosinase inhibitory effects, prevention of DOPA oxidation, and suppression of melanin production. The inhibition of tyrosinase and DOPA oxidation by LTEs was suggested to be related with the downregulation of microphthalmia-associated transcription factor, tyrosinase, tyrosinase-related protein-1, and tyrosinase-related protein-2, verified with mRNA and protein expression levels. Among all tested LTEs, 85% aq. MeOH and *n*-BuOH were found to be the most active fractions which later yielded the two known compounds, myricetin 3-galactoside and quercetin 3-*O*-β-galactopyronaside. The anti-melanogenic potential of the compounds were confirmed by their tyrosinase inhibitory effects. These results suggested that *L. tetragonum* may serve as a potential source of bioactive substances with effective anti-melanogenesis properties.

## 1. Introduction

Human skin has various defense mechanisms against environmental factors. Skin pigmentation through the production of melanin is one of the essential pathways for shielding skin tissue from radiation. However, overproduction of melanin and accumulation thereof are suggested to cause various dermatological complications, including freckles, spots, and other hyperpigmentation-related disorders [1]. Skin pigmentation is predominantly affected by genetic background. On the other hand, other non-genetic stimuli such as inflammation, hormonal changes, aging, and ultraviolet light are also known to enhance skin pigmentation through regulation of melanin-related pathways. These non-genetic factors are reported to affect skin pigmentation by stimulating the expression of three major enzymes involved in melanogenesis: tyrosinase, tyrosinase-related proteins-1 (TRP-1), and tyrosinase-related proteins-2 (TRP-2). Previous studies have reported that α-melanocyte-stimulating hormone (α-MSH), also known as the hormone melanocortin-1 (MC1), regulates the upregulation of microphthalmia-associated transcription factor (MITF) [2,3]. Binding of MITF to the gene promoters of melanogenic enzymes induces melanogenic gene expression. As a key mediator of melanogenesis, newly synthesized tyrosinase protein undergoes maturation and activation through multiple mechanisms including copper binding, glycosylation, and phosphorylation [4].

Melanin synthesis starts with the hydroxylation of l-tyrosine to DOPA and then to DOPA-quinone. These two reactions are catalyzed by the enzymatic activity of tyrosinase [5]. In the presence of TRP-2/DOPA-chrome tautomerase (TRP-2/DCT), DOPA-chrome is converted into 5,6-dihydroxy-indole-2-carboxylic acid (DHICA). TRP-1/DHICA oxidase catalyzes the oxidation of DHICA to indole-5,6-quinone-2-carboxylic acid. These two closely related structures, TRP-2/DCT and TRP-1, act to produce unstable quinones that undergo further polymerization, finally yielding brownish-black eumelanins [6]. In an effort to inhibit the activity of tyrosinase, many different types of tyrosinase inhibitors for preventing hyperpigmenation have been developed via either synthesis or isolation from natural sources [7].

Halophytes have been of much interest due to their known ability to withstand harsh environments by producing various secondary metabolites. Some halophyte species already have been reported to possess numerous health beneficial effects [8,9,10]. *Limonium tetragonum* is a common species of salt-tolerant halophyte that grows on salt marshes and muddy seashores throughout the western coastal area of South Korea. The literature does not contain any report on *L. tetragonum*’s potential health beneficial effects and bioactive constituents, except for its antioxidant activity which is common for most halophytes. Considering the potential halophytes hold, as a part of a prominent research program to develop novel substances from natural plants for nutraceutical and cosmetic purposes, *L. tetragonum* has been tested for its beneficial effects against melanogenesis and hyperpigmentation in vitro using B16-F10 mouse skin melanoma cells.

## 2. Results and Discussion

**Figure 1:**
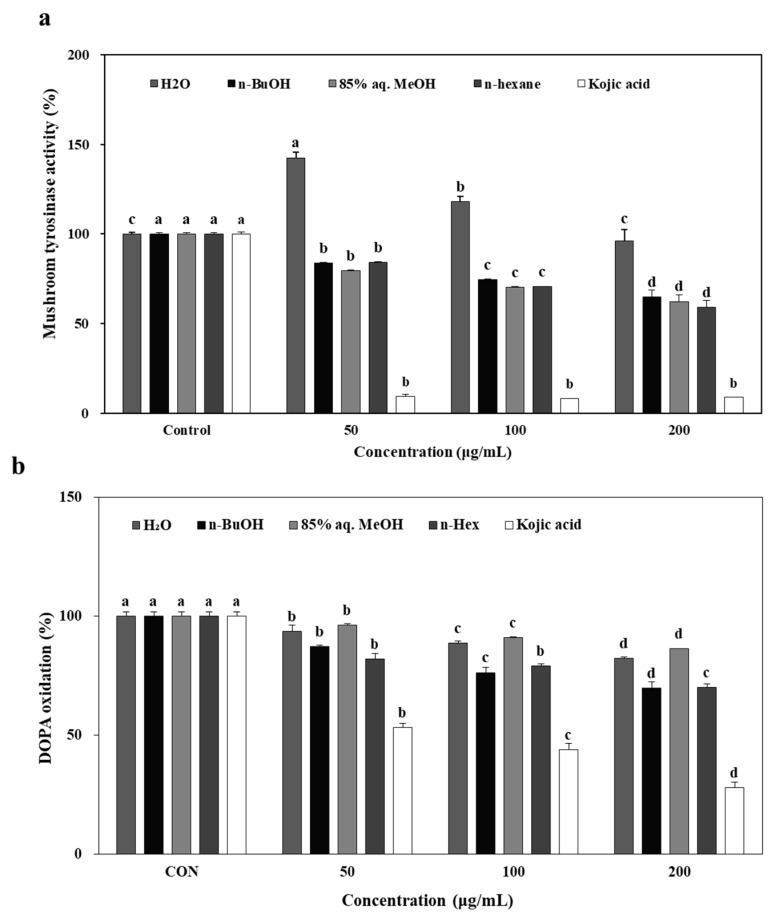
Effect of *L. tetragonum* solvent-partitioned fractions and kojic acid on mushroom tyrosinase activity (**a**) and DOPA oxidase activity of mushroom tyrosinase (**b**) in vitro. ^a–d^ Means with the different letters are significantly different (*p* < 0.05) by Duncan's multiple range test among same samples compared to control group.

The ability of *L. tetragonum* solvent-partitioned extracts (LTEs) to inhibit tyrosinase was tested on mushroom tyrosinase activity prior to DOPA oxidation, cellular tyrosinase activity and mechanism elucidation in B16-F10 cells. A dose-dependent, scientifically significant inhibitory effect for tested LTEs except H_2_O were observed on mushroom tyrosinase activity in vitro (Figure 1a). Kojic acid (200 μM), a reference compound which is a well-known melanogenesis inhibitor with potent tyrosinase inhibitory activity [11] was used a positive control. Despite the less significant inhibition compared to kojic acid, all tested samples except H_2_O LTE were found to possess potential to inhibit tyrosinase activity which lead to following anti-melanogenesis assays performed in in vitro model, B16-F10 mouse melanoma cells. In anti-melanogenesis studies, α-MSH is commonly used in the testing process. Melanin production is normally low if B16-F10 cells that are grown in the absence of α-MSH. In order to easily and clearly determine the influence of LTEs on cellular melanin formation, α-MSH was utilized to induce melanin production during the cell testing procedure. As reported earlier [12], α-MSH was used at 100 nM concentration which is an optimal minimum to stimulate melanogenesis.

The inhibition of the oxidation of DOPA to DOPA-quinone in the presence of LTEs, and Kojic acid (50, 100 and 200 μg/mL) for 72 h was presented compared to the control cells without any treatment (Figure 1b). The treatment with LTEs inhibited DOPA oxidase activity significantly, however, not as substantial as kojic acid which showed an inhibition ratio of 76% compared to control cells.

**Figure 2:**
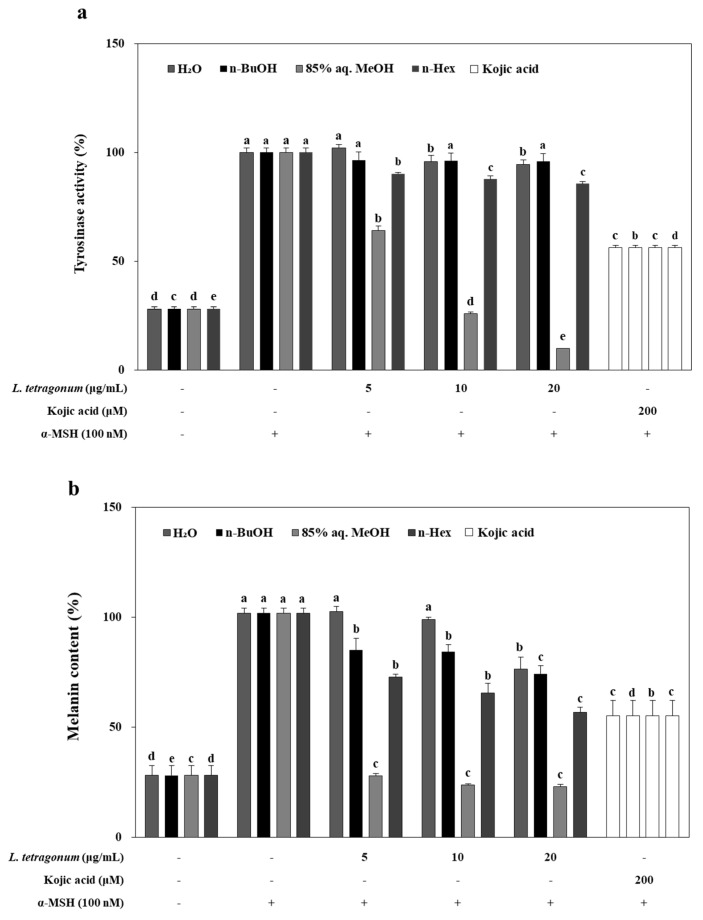
Inhibition of cellular tyrosinase activity (**a**) and reduction in melanin content (**b**) in B16-F10 cells by *L. tetragonum* solvent-partitioned fractions and kojic acid. ^a–e^ Means with the different letters are significantly different (*p* < 0.05) by Duncan's multiple range test among same samples compared to control group.

As presented in Figure 2a, inhibition of cellular tyrosinase activity in B16-F10 cells treated with LTEs (5, 10, and 20 μg/mL), and kojic acid (200 μM) for 72 h in the presence of 100 nM α-MSH produced notable results. Unlike DOPA oxidation inhibition results, *n*-BuOH LTE treatment inhibited the tyrosinase activity approximately by 19% of control group at the concentration of 20 μg/mL while kojic acid was able to inhibit the tyrosinase activity by 52%. Additionally, 85% aq. MeOH LTE inhibited the tyrosinase activity notably in a dose-dependent manner, by 38% and 97% compard to control at the concentrations of 5 and 10 μg/mL, respectively. Other tested LTEs were observed to present similar inhibitory efficiency that was exhibited in DOPA oxidation assay.

B16-F10 cells were exposed to LTEs (5, 10 and 20 μg/mL) in the presence of α-MSH (100 nM) for 72 h, and the extracellular melanin release was measured (Figure 2b). These results suggested that *n*-BuOH LTE suppressed α-MSH-induced cellular melanin biosynthesis by inhibiting tyrosinase activities rather than DOPA oxidase activities. Despite being less effective in inhibiting DOPA oxidation compared to the positive control kojic acid, but able to inhibit tyrosinase activity, 85% aq. MeOH LTE (20 μg/mL) decreased the melanin release approximately 77% of control cells while kojic acid was only able to lower this to 54% of control cells. Some reports have suggested that antioxidants may prevent or delay pigmentation by different mechanisms. These mechanisms were suggested as scavenging.

ROS and reactive nitrogen species, reduce the amount of *o*-quinones or other intermediates formed in melanin biosynthesis and delay oxidative polymerization. The antioxidant potential of halophytes, and *L. tetragonum* thereof, was reported by several studies [9,13,14,15]. It has also been reported that free radicals might enhance the expression levels of tyrosinase in melanin biosynthesis [16]. Hence, possible antioxidant constituents of LTEs may play an important role in inhibiting the tyrosinase activity and linked melanogenesis.

**Figure 3:**
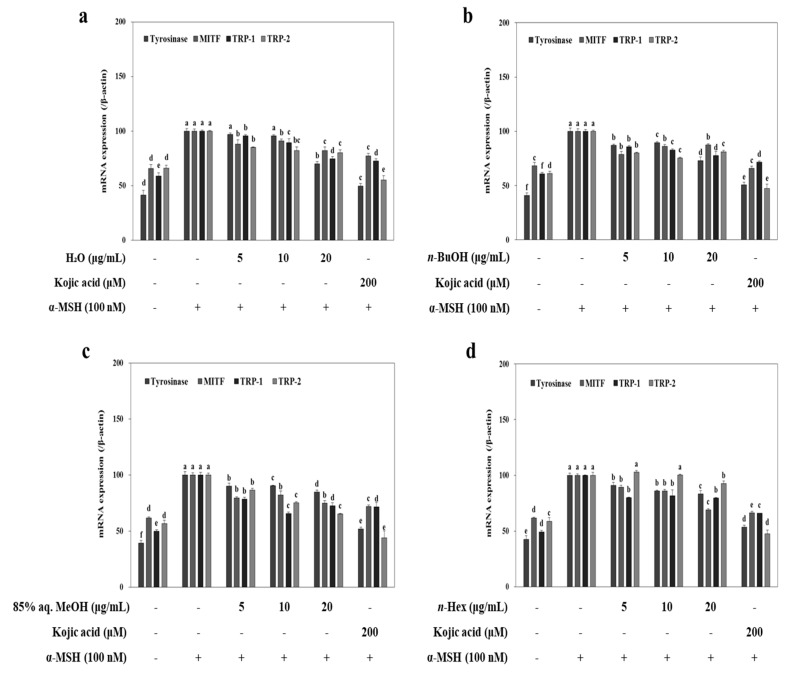
Changes in expression of tyrosinase, MITF, TRP-1, and TRP-2 mRNA levels in *L. tetragonum* solvent-partitioned fractions (**a**: H_2_O; **b**: *n*-BuOH; **c**: 85% aq. MeOH; **d**: *n*-Hexane) and Kojic acid treated B16-F10 cells. ^a–e^ Means with the different letters are significantly different (*p <* 0.05) by Duncan’s multiple range test among same samples compared to control group.

**Figure 4:**
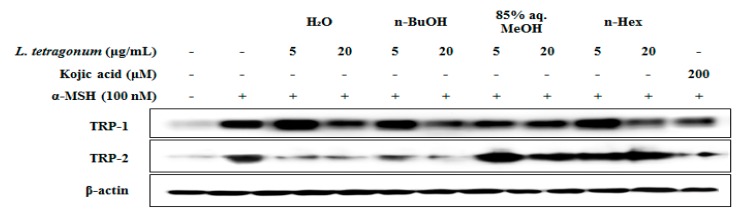
Changes in protein levels of TRP-1, and TRP-2 in *L. tetragonum* solvent-partitioned fractions and Kojic acid treated B16-F10 cells.

To elucidate the possible mechanism of the inhibition of melanin biosynthesis by LTEs, melanogenesis-related mRNA and protein levels, such as tyrosinase, MITF, TRP-2 and TRP-1 were evaluated. B16-F10 cells were exposed to α-MSH (100 nM) in the presence of LTEs (5, 10 and 20 μg/mL) for 72 h, and mRNA levels were examined by RT-PCR. Elevated mRNA levels following α-MSH stimulation were lowered by treatment of LTEs and kojic acid (Figure 3). Although the efficiency of 85% aq. MeOH LTE was not as high as its inhibition of melanin production, it provided the most similar inhibitory effect to that of kojic acid among all tested LTEs. Similar inhibition patterns were observed in protein levels of TRP-1 and TRP-2 after LTE treatment (Figure 4). Treatment with *n*-BuOH showed the highest decrease in the levels of TRP-1 and TRP-2 which further verifies the suggestion of tyrosinase-linked inhibition of melanogenesis by LTEs.

**Figure 5:**
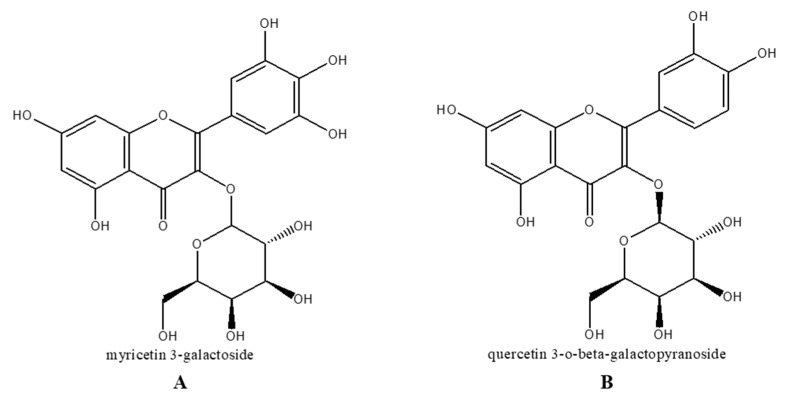
Isolated compounds from active *n*-BuOH fraction of *L. tetragonum*.

**Figure 6:**
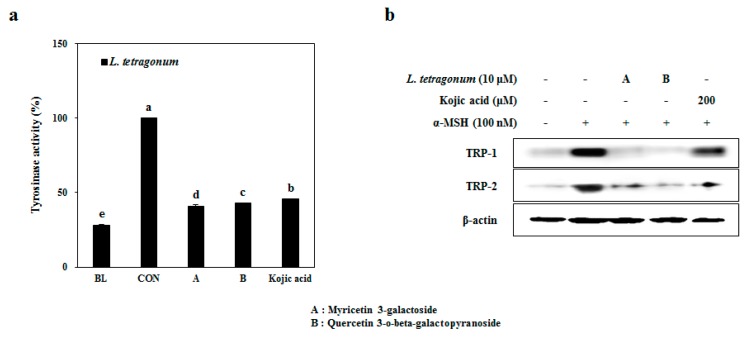
Inhibition of cellular tyrosinase activity (**a**) and protein levels of TRP-1 and TRP-2 (**b**) in B16-F10 cells by isolated compounds. ^a–e^ Means with the different letters are significantly different (*p* < 0.05) by Duncan’s multiple range test among same samples compared to control group.

Comparing the ability of LTEs to inhibit melanogenesis, through an activity-guided isolation two known compounds, myricetin 3-galactoside (A) and quercetin 3-*o*-beta-galactopyronaside (B) were isolated (Figure 5) as described earlier [17]. These two compounds are known derivatives of antioxidant and anti-melanogenic compounds which were previously reported [18,19,20]. In order to verify the compounds as active constituents of the LTEs, their inhibitory effects on tyrosinase activity were tested (Figure 6a). Expectedly, both compound A and B were able to lower the cellular tyrosinase activity by 65% and 63%, respectively, compared to 59% inhibition of tyrosine activity by Kojic acid treatment. Further, both compounds were able to decrease the protein levels of TRP-1 and TRP-2 significantly with an efficiency superior to that of kojic acid (Figure 6b) suggesting their involvement in regulating the tyrosinase activity through TRP-linked pathways. Possible antioxidant properties of these compounds [21] were suggested to be the underlying reasoning of their successful tyrosinase inhibitory effect. 

## 3. Materials and Methods 

### 3.1. Plant Material

*L. tetragonum* was provided by Korea Maritime and Ocean University (Yeongdo, Busan, Korea). The shade-dried material (500 g) of *L. tetragonum* was ground and sequentially extracted twice with CH_2_Cl_2_ (3 L) and MeOH (3 L), respectively. The combined crude extracts were dried in vacuo to obtain a residue of 32.06 g and partitioned between CH_2_Cl_2_ and H_2_O. The organic layer was fractionated with *n*-Hexane and 85% aqueous MeOH. The aqueous layer was further fractionated with *n*-BuOH and H_2_O, resulting in *L. tetragonum* solvent-partitioned fractions (LTEs) of *n*-hexane (2.64 g), 85% aqueous (aq.) MeOH (1.42 g), *n*-BuOH (1.53 g), and H_2_O (26.47 g). Fractions were dissolved in 10% DMSO in order to be used in experiments and stored at 20 °C. Further isolation of the active compounds were carried out as described earlier [15].

### 3.2. Cell Culture

Murine melanoma B16-F10 cells were purchased from the Korean Cell Line Bank (KCLB, Seoul, Korea). Cells were cultured in Dulbecco’s modified Eagle medium (DMEM, Gibco-BRL, Gaithersburg, MD, USA), which was supplemented with 10% fetal bovine serum (FBS, Atlas, Fort Collins, CO, USA) and 1% penicillin−streptomycin (Sigma-Aldrich, St. Louis, MO, USA) at 37 °C in 5% CO_2_ atmosphere.

### 3.3. Mushroom Tyrosinase Activity

Mushroom tyrosinase inhibitory activities of the samples were measured using a spectrophotometric method. Sample solution (10 μL) with different concentrations and mushroom tyrosinase (20 μL; 1000 units/mL) in 50 mM phosphate buffer (pH 6.5) were added to assay mixture (170 μL) in 96-well plates containing a ratio of 10:10:9 of 1 mM l-tyrosine solution, 50 mM potassium phosphate buffer (pH 6.5), and distilled water. Following a 30 min of incubation at 37 °C, the absorbance of the mixture was determined at 490 nm using a GENios microplate reader (Tecan Austria GmbH, Grödig, Austria). The extent of inhibition by addition of samples was expressed as the concentration required for a 50% inhibition (IC_50_). Calculation of percentage inhibition of mushroom tyrosinase activity was calculated by equation below:

Inhibition % = [{1 − (Aa − Ab)/Ac} × 100]

where Aa is the absorbance of the sample wells, Ab is the absorbance of the sample wells without enzyme, and Ac is the absorbance of the control wells with enzyme but without sample.

### 3.4. DOPA Oxidase Activity of muShroom Tyrosinase

The DOPA oxidase activity of mushroom tyrosinase was measured using a colorimetric assay, which relies on the oxidation of DOPA to DOPA-quinone. Assay mixtures (final volume of 140 μL) were prepared in tubes by dissolving 1 mg/mL LTE stock solutions (final concentrations of 50, 100, and 200 μg/mL) and kojic acid (final concentration of 200 μM) in 0.05 M sodium phosphate buffer (pH 6.8). Next, twenty μL of mushroom tyrosinase solution (2000 units/mL) was added to assay mixtures and incubated for 10 min at 37 °C. Reaction was started with addition of 40 μL of 6 mM 3,4-dihydroxy-l-phenylalanine (l-DOPA) to each well. The absorbance values were calculated after 15 min incubation at 37 °C at 490 nm using a microplate reader (Tecan Austria GmbH, Grödig, Austria). In control wells neither LTEs nor kojic acid (positive control) were included.

### 3.5. Cellular Tyrosinase Activity

Activity of tyrosinase in B16-F10 cells was evaluated by measuring l-DOPA oxidation rate. B16-F10 cells were plated in 6-well plates at a density of 5 × 10^4^ cells/well and incubated at least 24 h prior to use. The cells were stimulated by addition of 2 μL α-MSH from 100 μM stock solution (final concentration of 100 nM) alone or together with 10, 20, and 40 μL of 1 mg/mL LTE stocks (final concentrations of 5, 10 and 20 μg/mL of LTEs, respectively), and 16 μL of 25 mM Kojic acid (final concentration of 200 μM) for 72 h. Cells were washed twice with phosphate buffer saline (PBS) and lysed in 200 μL of lysis buffer (5 mM EDTA, 0.1 M sodium phosphate buffer (pH 6.8), 1% Triton X-100, 0.1 mM phenylmethylsulfonyl fluoride). Obtained lysates were centrifuged at 1000 *g* and the supernatant were used for tyrosinase activity assay. Protein quantification was performed by measuring the absorbance at 595 nm using Bio-Rad protein assay solution (Bio-Rad, Hercules, CA, USA) and calculating the amount of protein equivalent. Assay mixture was prepared by mixing the cell lysate supernatant and 0.1 M sodium phosphate buffer (pH 6.8) at a ratio of 1:3 to the total volume of 150 μL. Reaction was started with addition of 50 μL 0.1% l-DOPA solution (*w*/*v*) to assay mixture. Following an incubation period of 1 h absorbance values were recorded at 490 nm with a microplate reader (Tecan Austria GmbH). Tyrosinase activity was calculated as a percentage of non-treated control wells. Testing the inhibitory effects of isolated compounds A and B on cellular tyrosine activity in B16-F10 cells was carried out with same procedure by addition of the compounds at the concentration of 10 μM.

### 3.6. Melanin Content

Melanin content of the B16-F10 melanoma cells were assayed according to modified method of Hosoi et al. [22]. Briefly, cell lysates obtained in tyrosinase activity assay were washed with 75% cold ethanol and dried prior to dissolve in 200 μL 1 N NaOH in 1% DMSO (*w*/*v*). Melanin in the mixture was dissolved by hot water bath at 80–90 °C for 1 h. Absorbance values of the mixtures were measured by ELISA Reader (Multiskan GO, Thermo Scientific, Vantaa, Finland) at 405 nm. Melanin content was calculated with plotting against melanin standard solution (Sigma-Aldrich) absorbance values.

### 3.7. RNA Extraction and Reverse Transcription-Polymerase Chain Reaction Analysis

B16-F10 cells were plated in 6-well plates at a density of 5×10^4^ cells/well and incubated at least 24 h prior to use. The cells were stimulated by α-MSH (100 nM) alone or together 5, 10 and 20 μg/mL of LTEs, and kojic acid for 72 h. Total RNA was isolated from B16-F10 cells using Trizol reagent (Invitrogen, Carlsbad, CA, USA). For synthesis of cDNA, RNA (2 μg) was added to RNase-free water and oligo (dT), denatured at 70 °C for 5 min and cooled immediately. RNA was reverse transcribed in a master mix containing 1× RT buffer, 1 mM dNTPs, 500 ng oligo (dT), 140 U M-MLV reserve transcriptase and 40 U RNase inhibitor at 42 °C for 60 min and at 72 °C for 5 min using an automatic T100 Thermo Cycler (Bio-Rad, Watford, UK). The target cDNA was amplified using the gene-specific sense and antisense primers. The amplification cycles were carried out at 95 °C for 45 s, 60 °C for 1 min and 72 °C for 45 s. After 30 cycles, the PCR products were separated by electrophoresis on 1.5% agarose gel for 30 min at 100 V. Gels were then stained with 1 mg/mL ethidium bromide visualized by UV light using by Davinch-Chemi CAS-400SM Imager (Davinch-k, Seoul, Korea) and AlphaEase^®^ gel image analysis software (Alpha Innotech, San Leandro, CA, USA). Gel images of RT-PCR were quantified and normalized to β-actin levels.

### 3.8. Western Blot Analysis

B16-F10 cells were plated in 6-well plates at a density of 5 × 10^4^ cells/well and incubated at least 24 h prior to use. The cells were stimulated by α-MSH (100 nM) alone or together 5, 10 and 20 μg/mL of LTEs, and kojic acid for 72 h. Immunoblotting was carried out following the standard procedures. Lysates were prepared from B16-F10 cells using RIPA lysis buffer (Sigma-Aldrich) at 4 °C for 30 min. Lysates were subjected to gel (12% SDS) electrophoresis for separation, followed by transfer of separated proteins onto polyvinylidene fluoride membrane (Amersham Pharmacia Biotech., Little Chalfont, UK). Blocking of the membrane was achieved with 5% skim milk prior to hybridization with diluted (1:1000) primary antibodies. Proteins were detected after incubation with horseradish-peroxidase-conjugated secondary antibody at room temperature, by a commercial chemiluminescece ECL assay kit (Amersham Pharmacia Biosciences) according to the kit manual. Immunoreactive protein bands were observed with Davinch-Chemi CAS-400SM Imager (Davinch-k, Seoul, Korea).

### 3.9. Statistical Analysis

The data were presented as mean ± SD of three different experiments (*n* = 3). Differences between the means of the individual groups were analyzed using the analysis of variance (ANOVA) procedure of Statistical Analysis System, SAS v9.1 (SAS Institute, Cary, NC, USA) with Duncan’s multiple range tests. The significance of differences was defined at the *p* < 0.05 level.

## 4. Conclusions

In summary, the anti-melanogenic activities of *L. tetragonum* were verified by testing its solvent-partitioned fractions against melanin production, tyrosinase activity and DOPA oxidation as well as melanogenesis-regulating pathways. Among all tested samples, 85% aq. MeOH fraction of *L. tetragonum* was observed to inhibit the melanin content and cellular tyrosinase activity comparable to positive control while showing notable but not comparable activities against DOPA oxidation. Other tested samples were also efficient to a content except *n*-BuOH LTE which was able to inhibit DOPA oxidation. In conclusion, *L. tetragonum* was suggested to contain anti-melanogenic substances, including but not limited to myricetin 3-galactoside and quercetin 3-*o*-beta-galactopyronaside that can prevent hyperpigmentation through inhibition of tyrosinase activity and TRP-linked melanin biosynthesis. Detailed action mechanisms of isolated compounds and their efficiency in vivo remains unclear. Nonetheless, potential of *L. tetragonum* as a source of anti-melanogenic compounds for nutraceutical and cosmetic fields was suggested by current study and paved the way for detailed analysis in order to facilitate its potential.
